# A comparison of the effectiveness of azelaic and pyruvic acid peels in the treatment of female adult acne: a randomized controlled trial

**DOI:** 10.1038/s41598-020-69530-w

**Published:** 2020-07-28

**Authors:** Karolina Chilicka, Aleksandra M. Rogowska, Renata Szyguła, Iwona Dzieńdziora-Urbińska, Jakub Taradaj

**Affiliations:** 1 Faculty of Health Sciences, University of Opole, 45-060 Opole, Poland; 2 Institute of Psychology, University of Opole, 45-052 Opole, Poland; 3 Institute of Physiotherapy and Health Sciences, Academy of Physical Education, 40-065 Katowice, Poland

**Keywords:** Diseases, Skin diseases, Acne vulgaris

## Abstract

Chemical peels are widely used as therapeutic agents in dermatology and cosmetology. This study aims to explore the differences in the effectiveness of azelaic and pyruvic acid peels in the treatment of acne vulgaris. Eligibility criteria for participants were: female gender, 18–25 years of age, no dermatological treatment within the last 12 months and mild to moderate papulopustular acne. We treated 120 young women (with a mean age of 22 years old) with six peeling sessions at 2-week intervals. In the parallel clinical study design, one randomized group (*n* = 60, 50%) was treated using azelaic acid (AA), whereas the second group participated in pyruvic acid (PA) sessions. We evaluated the patients clinically twice (before and after treatment), using the Scale of Hellegren–Vincent Severity Symptoms to assess the acne diagnosis, and the Nati Analyzer to estimate the skin properties (oily skin, desquamation, porosity, and moisture). The clinical evaluation of the patients demonstrated a significant reduction of acne severity symptoms in both the AA and PA groups, after the peeling sessions. An effect was also found in terms of decreasing desquamation and the oiliness of the skin. PA showed a more significant reduction of greasy skin than AA. In conclusion, after the six peeling sessions using AA and PA, all patients showed better skin parameters in term of reduced oiliness and desquamation. Both AA and PA peelings are a safe and efficient treatment for mild acne, however, during the selection of one of the two acids, side effects, skin properties, and patients’ preferences should be taken into account. This study was registered in the ISRCTN registry (registration number ISRCTN79716614, 17/01/2020).

## Introduction

### Characteristics of acne vulgaris

Acne vulgaris is a chronic inflammatory disease of the skin, that is characterized by comedones, seborrhea, nodules, papules, pustules, and scarring. The sebaceous glands are formed as a result of elevated testosterone levels, follicular hyperkeratinization, and excessive colonization of Cutibacterium acnes (old name *Propionibacterium acnes*-*P. acnes*), which leads to immune reactions and as a consequence to inflammation^1,2^. The prevalence of acne vulgaris is widespread, with 95% of boys and 83% of girls at 16 years old experiencing it. Females suffer from acne vulgaris at an earlier age in comparison to males, most likely, this is a result of the earlier onset of puberty among females^3^. Although acne tends to be more persistent in females, males usually experience more severe forms of this disease. Furthermore, the locations of acne differ for both genders, with acne occurring more frequently on the face for women while being more prevalent on the chest and back for men^4^.

Adult acne (acne trada) is most common in female individuals ranging between 20 and 25 years of age. There are two subtypes of acne trada: late acne (also knowns as persistent, when emerging in adolescence and continuing to adulthood; accounting for 80% of cases) and late-onset (if first presenting in adulthood). Adult acne differ from adolescent type^5^. The inflammatory form of adult acne is characterized by papulopustular and deep inflammatory nodules, predominating on the neck, jaw and chin. The comedonal form consists of macrocomedones (micro cysts). The most common type of female adult acne is mild to moderate and usually is refractory to treatment^5,6^.

### Treatment of acne

Topical treatment is used most frequently for acne patients. In particular, topical maintenance treatment is recommended after treatment discontinuation in adult female acne, to decrease the risk of acne relapse^3^. Concurrently, systemic drug therapy may also be included, depending on the severity of the disease. Because the pathogenesis of acne is multifactor, treatment should comprise various methods: topical photodynamic therapy, topical retinoids, azelaic acid, benzoyl peroxide, topical and oral antibiotics, oral isotretinoin, hormonal therapy, insulin-sensitizing agents, 5α-reductase type 1 inhibitors, low-dose long-term isotretinoin regimens, anti-inflammatory agents such as lipoxygenase, and a special diet. One of the most efficacious methods to reduce acne scars is chemical peeling^7,8^.

The chemical peeling of facial skin is a useful method in modern cosmetology for the resurfacing of aging and sun-damaged skin, as well as to treat various skin diseases^9^. According to the definition of the American Academy of Dermatology, “chemical peeling (chemexfoliation) for the treatment of certain cutaneous diseases or conditions or aesthetic improvement, consists of the application of one or more chemical exfoliating agents to the skin, destroying portions of the epidermis or dermis and the regeneration of new epidermal and dermal tissues.”^10^ One or more exfoliating agents are used on the epidermis and derma skin at well determined times^4^. Chemical peeling aims to remove damaged facial skin in a controlled manner in order to smooth and improve its texture. This effect is also achieved by stimulating a wound-healing response^9^. The dermis thickens as a result of increasing growth factors and collagen production caused by chemical injury.

### Clinical effect of azelaic and pyruvic acid peels on acne

Peeling is one of the oldest and most popular cosmetic procedures worldwide^11^. Superficial chemical peels, also called “refreshing peels” or “light peels”, are defined by the application of one or more agents to the skin with the aim of mild desquamation. The alpha-hydroxy acids (AHAs) are a group of organic compounds extracted from fruit and sugarcane that have a hydroxyl in the alpha position. AHAs are metabolites of the carbohydrate cycle and other critical metabolic processes. For medium peels, the azelaic AHAs stand out as having the highest efficacy in improving the quality and appearance of facial skin, especially in the reduction of papular and pustular acne^4,11^.

Pyruvic acid (PA, CH_3_–CO–COOH) is an α-keto-acid which has gained significant attention in recent years because of its various keratolytic, antimicrobial, and sebostatic properties as well as its ability to stimulate the formation of new collagen and elastic fibers^8,11^. PA converts physiologically to lactic acid, and its properties make it a particularly effective topical peeling agent, with a low risk of scarring. PA causes dermo-epidermal separation and increases the production of collagen, elastic fibers, and glycoproteins, as well as demonstrating antimicrobial activity. Following its keratolytic and desmoplastic properties, PA has been employed as a medium peeling agent in subjects with inflammatory acne, moderate acne scars, greasy skin, actinic keratosis, and warts. Apart from being useful for acne, photodamage, and superficial scarring, the agent has also shown benefit in several pigmentary disorders in light-skinned patients^11,12^.

Azelaic acid (AA) is a naturally occurring saturated C9-dicarboxylic acid, which is effective in the treatment of acne^13^. It has shown anti-inflammatory and antibacterial properties against acne bacteria^14,15^. The antibacterial action affects different cutaneous microorganisms, inhibiting the synthesis of cellular protein in aerobic and anaerobic microorganisms, such as *P. acnes* and *S. epidermidis*^1,16^. Furthermore, AA inhibits free radical production with neutrophils, especially in melasma and post-inflammatory hyperpigmentation. Clinical research reveals that AA has excellent effectiveness in the treatment of acne vulgaris, does not cause complications, and is well-tolerated by most patients^2,15,17,18^.

### The present study

Although both AA and PA acids are well-known in acne treatment, there is insufficient knowledge regarding whether the efficacy of these acids is similar or significantly different. This study compares AA and PA to examine their effectiveness in the treatment of acne vulgaris in young adult women. To the best of our knowledge, we are exploring the efficacy of the two acne treatments for the first time; thus, we did not formulate any specific hypotheses.

## Results

**Figure 1:**
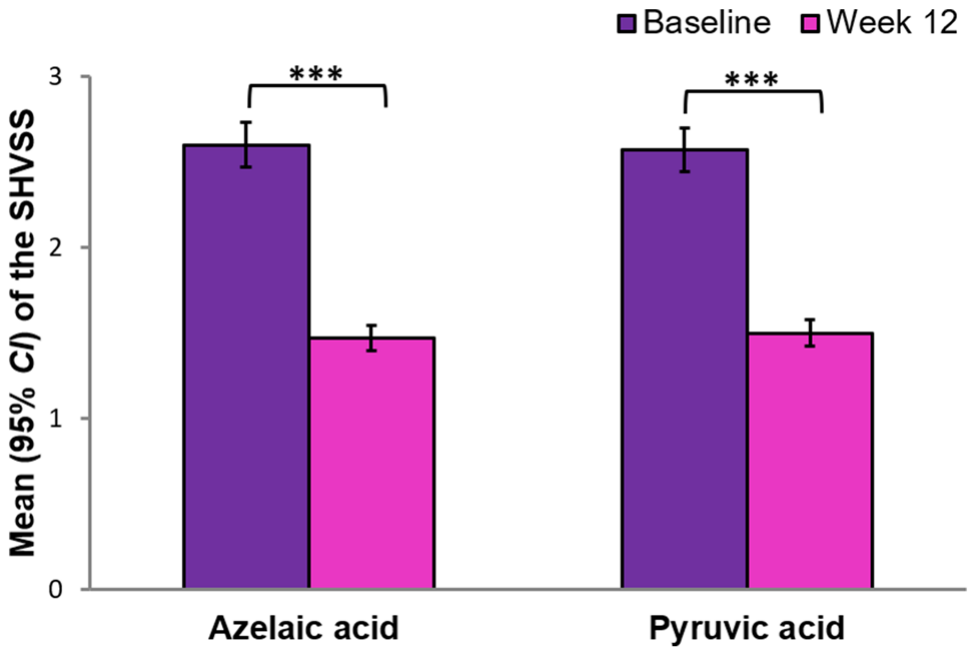
Mean scores of the Scale of Hellegren–Vincent Severity Symptoms (SHVSS) at baseline and 12 weeks after treatment with azelaic or pyruvic acid. The error bars are the 95% confidence interval (*CI*). ***p < .001.

**Figure 2:**
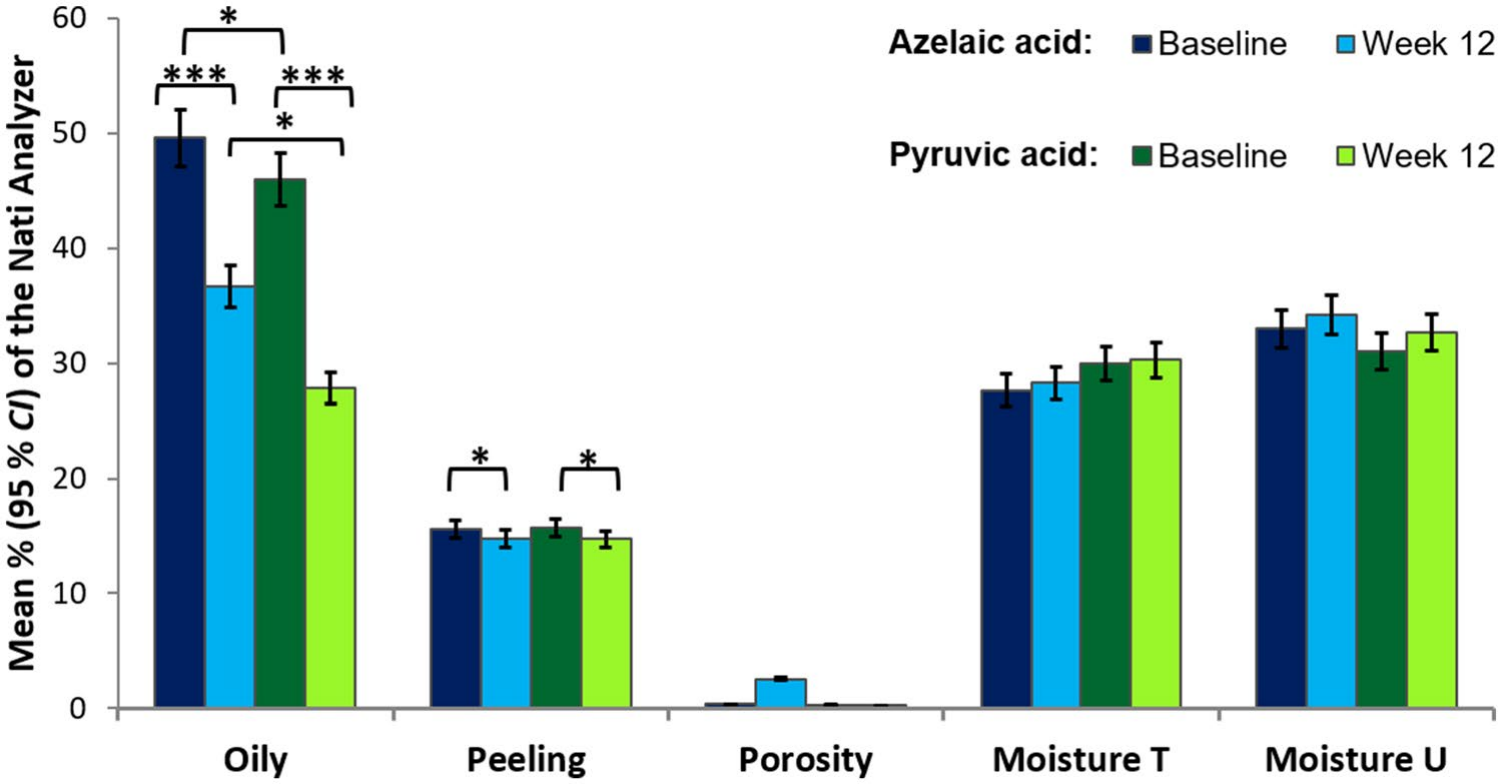
Mean percentages of the Nati Analyzer for oiliness, peeling, porosity, moisture T and moisture U at baseline and 12 weeks after treatment with azelaic or pyruvic acid peels. The error bars are 95% *CI*. *p < .05; **p < .01; ***p < .001.

The number of participants who were randomly assigned and received the acid peels was 60 in both AA and PA groups. There were no cases of losses and exclusions after randomization. The mean results, standard error, and 95% confidence interval (*CI*) are shown in Table 1. Table 2 shows the results of the one-way ANOVA with repeated measures. The differences in the SHVSS between the AA and PA groups are shown in Fig. 1. According to the assumptions, the degree of acne severity symptoms in the SHVSS decreased significantly under the treatment of both acids AA and PA (*p* < 0.001) (Fig. 2). The effect size estimated by η_*p*_^2^ for the main effect of Treatment (Test, Retest) was very large and was able to explain about 80% of acne variance. There were no significant differences between AA and PA acid peels in the treatment of acne.

**Table 1:** Comparison of the mean scores for skin parameters during 12 weeks of azelaic and pyruvic acid treatment.

Parameters	Before treatment			After treatment		
	M	SE	95% CI	M	SE	95% CI
**Pyruvic acid group (n = 60)**						
Oiliness	45.94	2.49	[41.02, 50.86]	27.85	2.82	[22.26, 33.43]
Peeling	15.66	0.23	[15.20, 16.11]	14.72	0.34	[14.04, 15.39]
Porosity	0.31	0.01	[0.30, 0.33]	0.23	1.61	[− 2.96, 3.42]
Moisture T	29.95	1.44	[27.11, 32.79]	30.28	1.35	[27.62, 32.95]
Moisture U	31.03	1.15	[28.76, 33.31]	32.68	1.17	[30.37, 35.00]
SHVSS	2.60	0.06	[2.47, 2.73]	1.47	0.07	[1.34, 1.60]
**Azelaic acid group (n = 60)**						
Oiliness	49.58	2.49	[44.66, 54.51]	36.72	2.82	[31.13, 42.30]
Peeling	15.60	0.23	[15.15, 16.05]	14.75	0.34	[14.07, 15.42]
Porosity	0.35	0.01	[0.33, 0.36]	2.56	1.61	[− 0.63, 5.75]
Moisture T	27.65	1.44	[24.81, 30.49]	28.30	1.35	[25.63, 30.97]
Moisture U	33.02	1.15	[30.74, 35.29]	34.22	1.17	[31.90, 36.53]
SHVSS	2.57	0.06	[2.44, 2.69]	1.50	0.07	[1.37, 1.63]

**Table 2:** Results of one-way ANOVA with repeated measures before and after treatment using one of the two types of acid: pyruvic or azelaic.

Variables	*df*	F	*p*	η_p_^2^
Oiliness (A × T)	1,118	1.55	0.22	0.01
Acid (Pyruvic × Azelaic)	1	4.02	0.05	0.03
Treatment (Test × Retest)	118	54.24	0.00	0.31
Peeling (A × T)	1,118	0.02	0.88	0.00
Acid (Pyruvic × Azelaic)	1	0.00	0.96	0.00
Treatment (Test × Retest)	118	11.85	0.00	0.09
Porosity (A × T)	1,118	1.01	0.32	0.01
Acid (Pyruvic × Azelaic)	1	1.08	0.30	0.01
Treatment (Test × Retest)	118	0.88	0.35	0.01
Moisture T (A × T)	1,118	0.04	0.85	0.00
Acid (Pyruvic × Azelaic)	1	1.44	0.23	0.01
Treatment (Test × Retest)	118	0.35	0.56	0.00
Moisture U (A × T)	1,118	0.08	0.78	0.00
Acid (Pyruvic × Azelaic)	1	1.52	0.22	0.01
Treatment (Test × Retest)	118	3.12	0.08	0.03
SHVSS (A × T)	1,118	0.43	0.51	0.00
Acid (Pyruvic × Azelaic)	1	0.00	0.00	0.00
Treatment (Test × Retest)	118	467.28	0.00	0.80

The percentage of oiling and desquamation of the skin significantly decreased as a result of treatment with acids (Fig. 2). The effect size estimated by η_*p*_^2^ for the main effect of the Treatment (Test, Retest) was large for oily skin (and could explain about 31% of variance) and medium for peeling skin (with 9% of the total variance explained). The group treated with PA showed significantly less oily skin compared to the group treated with AA, but the effect size was small (with 3% of the total variance explained). Other indicators regarding pore size and skin moisture did not change significantly. There was no interaction between the groups and acid therapy.

## Discussion

### The efficacy of azelaic and pyruvic acid peels in reducing acne symptoms

The study found that AA and PA produced similar decreases in acne severity and lesions. The mean difference in the SHVSS between the baseline and week 12 of the acids treatment indicates that using either AA or PA may reduce acne symptoms. The mean overall global improvement was about 40% in both treatment groups. The present result is consistent with previous research. The effectiveness of acids has been confirmed in numerous studies^4,8,11,19^. Szymańska et al.^20^ showed that after a series of six acid treatments, performed every 2 weeks, there were reduced facial acne lesions and the activity of the sebaceous glands was normalized. Recent research indicate that pyruvic acid has a much better effect on the treatment of acne vulgaris, than a mixture of glycolic and salicylic acid^21^. Pyruvic acid showed a significant increase in skin hydration, and there was a decrease in the amount of melanin in the epidermis compared to the mixture of glycolic and salicylic acid, where the changes were not statistically significant. However, previous research did not found any differences in the efficacy of pyruvic and salicylic acid^22^. Both pyruvic and salicylic acid had similar effects, since the number of reduced skin eruptions was similar in both groups of patients.

Although the exact mechanism remains unclear, antimicrobial, anti-inflammatory and comedolytic modes of action seem to fulfill a vital role in acne treatment^15^. AA and PA may interfere with the transmembrane pH gradient, which mainly inhibits the protein synthesis of the susceptible microorganism. It was found that the reduction of the intrafollicular Cutibacterium acnes population possibly further produces a reduction of free fatty acids arising from triglycerides due to the action of bacterial lipases.

### A comparison of changes in skin parameters under the influence of AA and PA peelings

Both AA and PA produced a significantly lower desquamation level (by about 1% at week 12). The other skin parameters, including pore size, as well as moisture U and T level, did not change significantly under the influence of AA and PA. However, significant differences between these two agents were shown in the extent of the oily skin level. PA tended to reduce oiliness to a greater extent (by about 19% at week 12) than AA (by about 13% at week 12).

The effect of AA in sebum production remains unclear. Most patients report a gradual reduction in skin greasiness. In contrast, the results of the research are inconsistent and seem to depend on the method of treatment, and the combination of acid with the other cosmetic agents^2^. On the other hand, the excellent sebostatic properties of PA have been well-documented in previous studies^19,23^. Because of its small dimension, PA penetrates more rapidly and deeply through the skin than AA. However, the depth of penetration also depends on the PA concentration, friction, vehicle, passes, and exposure time^24^.

AA has an effective antibacterial action. It decreases the size and number of comedones by altering follicular hyperkeratosis as well as reducing post-inflammatory hyperpigmentation due to its anti-tyrosinase activity. Moreover, AA is neither toxic nor phototoxic and does not interact with other drugs, thus may be used during pregnancy and lactation^15,23^. Side effects of AA are not typical and include itching, burning, irritation, dysesthesia, allergic reactions toward the vehicle, tightness of the skin in the treated area, a mild bleaching effect and the aggravation of already inflamed skin^15,24^. The AA can be combined with hormonal contraceptives and, in severe cases of acne, with oral tetracyclines, to promote more rapid improvement^16^. There is consensus among dermatologists to recommend AA as a second-line therapy^24^.

However, PA was also found to be an effective and safe peeling agent that can improve skin texture and skin color and reduce active acne (especially microcystic acne) and hyperpigmented lesions^5^. PA also have well-known keratolytic, antimicrobial, and sebostatic properties, and stimulates the formation of collagen and elastic fibers. PA causes intense stinging and burning sensations during the application and produces pungent and irritating vapors for the upper respiratory mucosa^8,11,25^. Side effects also include crusting in areas of inflamed or thinner skin. For up to 6 months after peeling, some patients may experience tingling or burning sensations in the periorificial area, or even temporary hyperpigmentary side effects^26^.

Contraindications include herpes simplex virus infection, autoimmune skin disorders, pregnancy, isotretinoin treatment in the previous 3 months, and keloids and hypertrophic scars. Because of its deep and fast action, PA is not recommended for skin with a disrupted barrier such as ongoing dermatitis, retinoid irritation, seborrheic dermatitis, atopic dermatitis, or perioral dermatitis^24^. Despite these limitations, pyruvic acid is suggested as a useful agent in the acceleration of the efficacy of topical and systemic acne therapy, especially for oily skin and mild acne scars^27^.

### Limitations of the study and further directions of research

The results of this study are promising but require confirmation in further research. The first limitation of the study may be measurement; in the future, we would like to use more precise measuring tools (for example, DermaUnit SSC 3, Courage Khazaka Electronic). A specialist camera could be used to improve the test quality of the study. The Visiopor R PP 34 camera uses a specific UV-light to visualize the fluorescing acne lesions of an area of, at minimum, 8 × 6.4 mm. The orange–red fluorescence indicates the presence of *P. acnes* bacteria within clinically non-evident (follicular impactions and microcomedones) and clinically evident (comedones, papules, and pustules) lesions. The other limitation of the study is the research sample in terms of to gender and age; the study focused on a homogeneous group of young women, and so results cannot be generalized to men, adolescents and older people. In the future, a larger sample size should participate in the study, including both women and men with acne, as well as people without any skin problems or disease (for comparison). Furthermore, we would like to examine adolescents, as well as adults above 30 years old with acne.

## Conclusions

The effectiveness of azelaic and pyruvic acids in acne treatment is comparable, as confirmed in this study. Both acids produced similar decreases in acne severity and lesions and decreased desquamation. However, PA reduced greasy skin to a greater extent than AA. The selection of patients is mandatory when choosing between AA and PA peels. An acid treatment should be related to the skin structure, the expected effect on the oiliness of the skin, and the extent to which side effects occur. The sense of safety in women during hormonal therapy, pregnancy, and lactation should be prioritized^28^.

## Methods

### Participants

**Figure 3:**
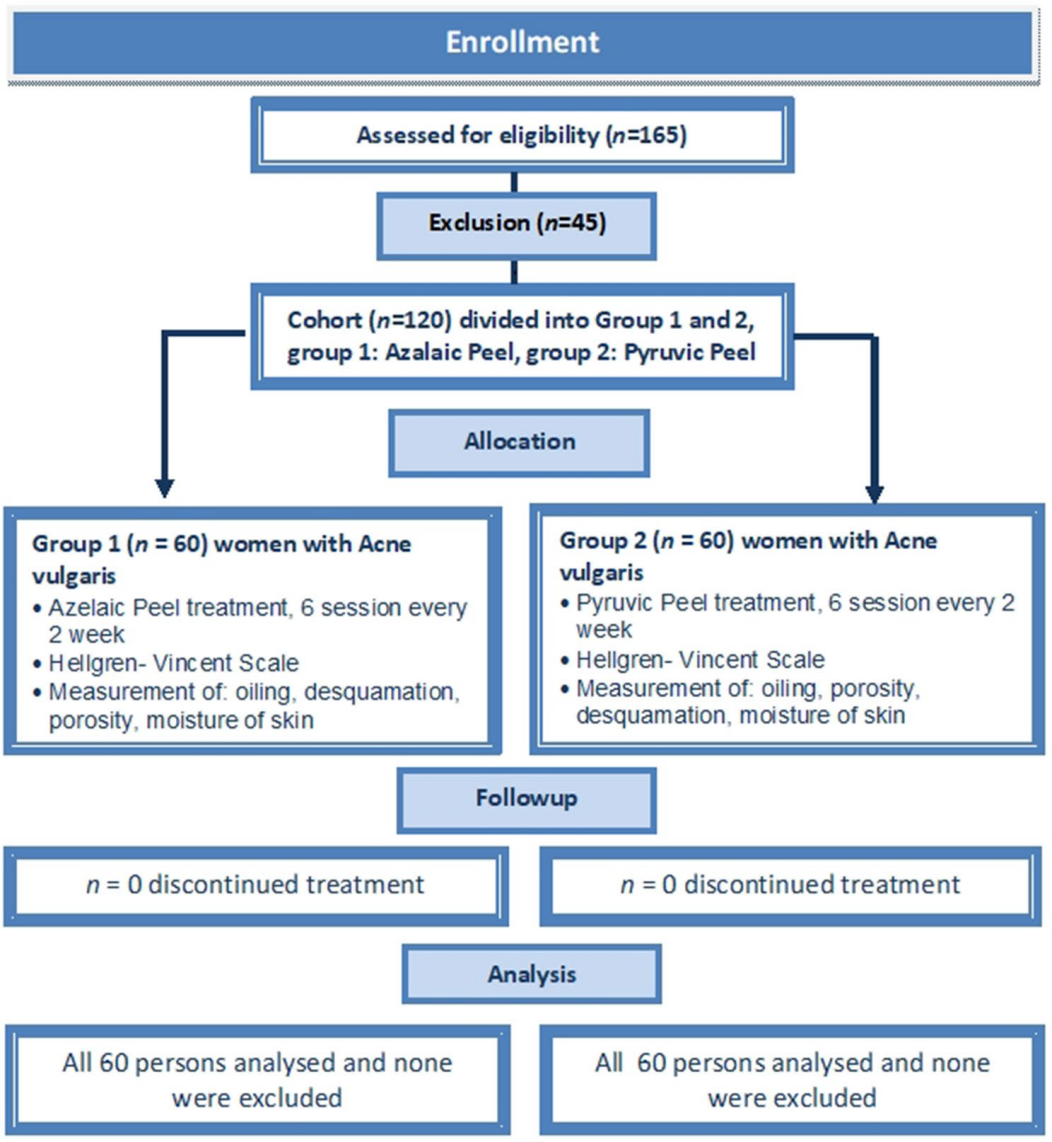
Consort flow chart of clinical study Group 1 and Group 2.

The study was designed as a prospective parallel randomized clinical trial. The sample size was determined using the Cohran formula with correction for the small population. Our initial estimate of sample size included an assumption of acne vulgaris occurrence among young adult women of 60%. We estimated that a total of 165 female students would be needed to detect a difference between groups, supposing an α of 0.05 (a 95% confidence level and 5% precision), out of a total population of 300 undergraduates studying Cosmetology at the university. The study initially recruited (between 27 and 31 January 2020) 165 female students; however, 45 of them did not fulfil the above criteria and they were excluded from the further study. Finally, the study sample included 120 women aged between 20 and 24 years old (*M* = 22.20, *SD* = 1.61), and they all were undergraduate students of third-year cosmetology at Opole Medical School in the south of Poland (Fig. 3).

Researcher Karolina Chilicka performed a random allocation sequence, enrolled participants, and assigned participants to interventions. The block randomization was used here to divide undergraduates into one of two parallel groups (AA, PA, AA, PA) in a 1:1 ratio. We divided the list of sequentially numbered students into odd numbers and even numbers; and then, the odd numbers were assigned to the AA group, while the even numbers were assigned to the PA group. Both AA and PA groups consisted of 60 women (50% of the total sample). Eligibility criteria were as follows: female gender, 18–25 years of age, no dermatological treatment within the last 12 months and mild to moderate papulopustular acne. The exclusion criteria were as follows: pregnancy, lactation, active inflammation of the skin, bacterial, viral, allergic and fungal relapsing skin diseases, disturbed skin continuity, new surgical procedures in the treatment area, active herpes, treatment with isotretinoin, reduced immunity, and allergy to peeling ingredients, active rosacea, eczema, psoriasis, numerous telangiectasias, numerous melanocytic nevi, tanned skin, skin cancers, autoimmune diseases such as pemphigus and collagenosis, recently having surgery (up to 2 months), recently having cryotherapy (up to 6 months), severe acne and propensity to keloids.

**Figure 4:**
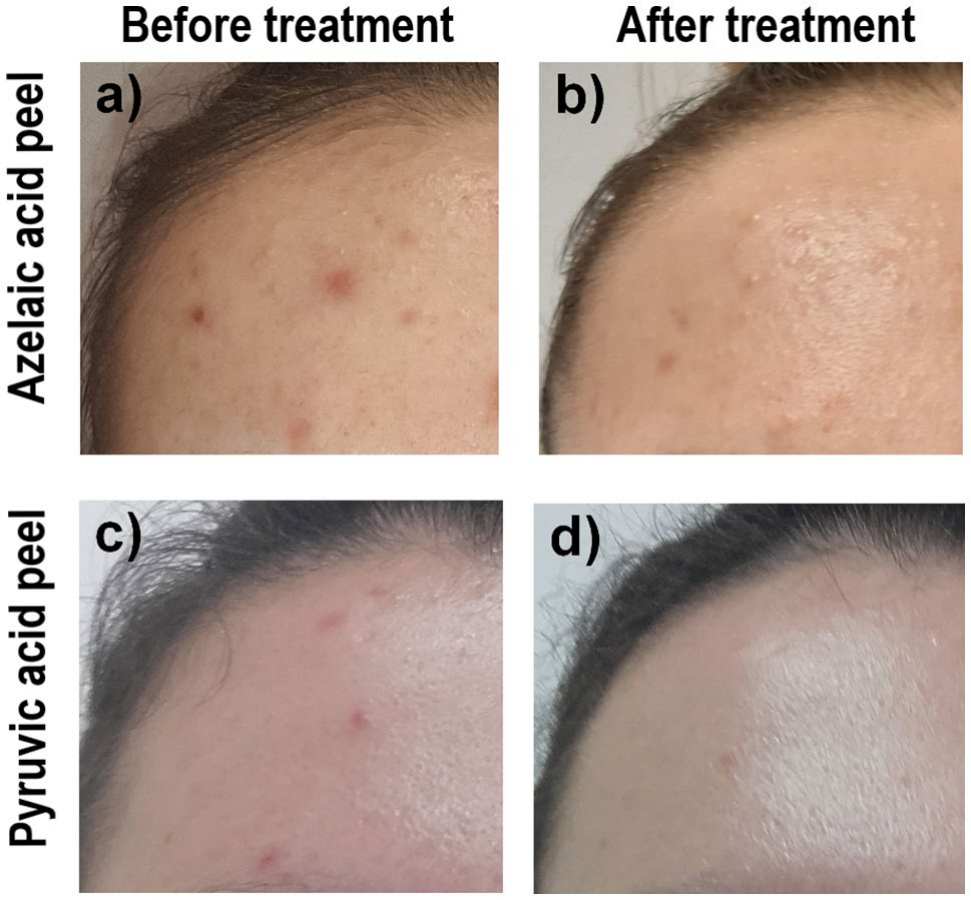
Example participant: (**a**) azelaic acid (AA) group, before treatment; (**b**) AA group, after treatment (**c**) pyruvic acid (PA) group, before treatment; (**d**) PA group, after treatment.

All participants presented with acne vulgaris, according to diagnosis using the Scale of Hellegren–Vincent Severity Symptoms (SHVSS). The SHVSS allows for the assessment of the overall severity of major acne symptoms, such as erythema, blackheads, pustule. The m and inflammatory papules (Fig. 4). This tool is useful for estimating the number of imperfections (papules, pustules, blackheads) and assessing the level of seborrhea. The mean degree of the SHVSS was 3 (*M* = 2.58, *SD* = 0.5; ranged between 2 and 3). Among participants, 50 demonstrated the second degree of the SHVSS (41.67% of total sample) and 70 showed the third degree (58.33%). There were not significant differences between AA and PA samples in severity of acne, χ(1)^2^ = 0.14, *p* = 0.71, ϕ = -0.03. The distribution of second-degree acne severity was similar in AA (*n* = 26, 43.33%) and PA (*n* = 24, 40.00%) samples. The third degree of acne severity prevailed in the AA (*n* = 34, 56.66%) as well as in PA (*n* = 36, 60.00%) groups. Among participants, 100% presented with mild or moderate type of late acne, with an average duration of acne persistence of 7 years (*M* = 6.78, *SD* = 0.64).

### Measures

Clinical evaluations were performed by using the Scale of Hellegren–Vincent Severity Symptoms (SHVSS). This tool is useful for estimating the number of imperfections (papules, pustules, blackheads) and assessing the level of seborrhoea. There are five degree of symptoms severity: (1) erythema, blackheads, 1–5 pustules or papules; (2) erythema, blackheads, 6–10 pustules or papules; (3) erythema, blackheads, 11–20 pustules or papules; (4) erythema, blackheads, 21–30 pustules or papules; (5) erythema, blackheads, over 30 pustules or papules. Functional measurements of the skin were performed before the procedure and 14 days after the complete session of four treatments, using the Nati Skin Analyzer device (Beauty of Science, Wroclaw, Poland). Using this device, we measured the sebum, the moisture of the T and U zone, exfoliation and the pore size of the skin.

The Nati Analyzer (NA) was used in this study for a comprehensive diagnosis of the following skin parameters: skin structure, desquamation level, moisture level, oily skin level, and pore size. The NA is a modern device in computer cosmetology diagnostics, which uses a digital camera operating HD Ready technology, and a 2-in-1 measurement system to allow a physical and optical analysis of the skin. The measurement was conducted twice, at baseline (before treatment), and 12 weeks later (14 days after six sessions with treatments using the acid peelings).

### Procedure

Six peel sessions were performed, each once every 2 weeks. Firstly, the face was prepared for the acid peeling by cleaning it using Pre Peel Cleanser, and then the skin was defatted with Pre Peel Lotion, for all of the participants. In the AA group, Azelaic Peel 1 (16% AA, 10% Almond acid, and 2% salicylic acid) was applied twice with a swab. Afterwards, Azelaic Peel 2 (16% AA) was twice applied with a swab. Each acid layer was applied after the previous acid layer had dried. The participants were instructed to leave the acid on the skin for 6–8 h, and then to wash their face. In the PA group, Pyruvic Peel (50% PA, and 50% pH 0.8) was applied with a cotton baguette three times for approximately one minute until erythema appeared. Next, the neutralizer (5 ml of pH indicators) was applied to the skin for 1 to 2 min. At the end of the session, protective cream with a 50 + UV filter was applied to the skin of the face home care was based on washing, removing make-up with micellar liquid, and using regenerating cream. During the 12 weeks of the peel sessions, none of the patients were treated with any other dermatological agents or cosmetological, and device treatments, apart from AA or PA peelings. No harm or unintended effects related to the treatment were reported by participants.

This prospective parallel clinical study with follow-up analysis was conducted between January and April 2020 at Opole Medical School, Opole, Poland. It was approved by the Human Research Ethics Committee of the Opole Medical School and conducted according to the principles of the Declaration of Helsinki (No. BC 1/2018). All participants provided their informed consent to participate in the study. Informed consent was also obtained for publication of identifying images in an online open-access publication. The Opole Medical School provided financial support for the study protocol registration. The study was registered in the International Standard Randomised Controlled Trial Number (ISRCTN) registry (registration number ISRCTN79716614, 17/01/2020). The subjects were informed that they could withdraw from the examination at any time, without giving a reason. Written, informed consent was obtained from all participants. The data for this paper are available in the Mendeley Datasets at https://dx.doi.org/10.17632/syft7bhs5n.2. The study protocol is available in the ISRCTN registry at https://www.isrctn.com/ISRCTN79716614.

### Statistical analysis

The repeated measures ANOVA was performed to examine differences between the AA and PA peels treatments. The dependent variable was the severity of acne symptoms (assessed by the SHVSS). The changes in skin parameters after acid peel treatments were also under control. The ANOVA with repeated measures was conducted separately for such skin parameters (as a dependent variable), as oiliness, peeling, porosity, moisture T and moisture U. Independent factor variables in all statistical analyses were Acid peels (Pyruvic, Azelaic) and Treatment time (Test = at baseline, Retest = after 12 weeks). In all of the following analyses, the effects of the treatment over time for the comparison groups (AA and PA) were examined in a two-tailed test, because we had no direct hypotheses. The Tukey’s honest significant difference (HSD) post-hoc test was conducted to find means that were significantly different from each other. The conditions’ effect sizes were calculated using the partial eta square (η_*p*_^2^).
